# Reassessment of Relevance and Predictive Value of Parameters Indicating Early Graft Dysfunction in Liver Transplantation: AST Is a Weak, but Bilirubin and INR Strong Predictors of Mortality

**DOI:** 10.3389/fsurg.2021.693288

**Published:** 2021-11-16

**Authors:** Margot Fodor, Adriana Woerdehoff, Wolfgang Peter, Hannah Esser, Rupert Oberhuber, Christian Margreiter, Manuel Maglione, Benno Cardini, Thomas Resch, Annemarie Weissenbacher, Robert Sucher, Heinz Zoller, Herbert Tilg, Dietmar Öfner, Stefan Schneeberger

**Affiliations:** ^1^Department of Visceral, Transplantation and Thoracic Surgery, Medical University of Innsbruck, Innsbruck, Austria; ^2^Department of Visceral, Transplantation, Thoracic and Vascular Surgery, University of Leipzig, Leipzig, Germany; ^3^Department of Internal Medicine I, Medical University of Innsbruck, Innsbruck, Austria

**Keywords:** liver, transplantation, early allograft dysfunction, outcome, reassessment

## Abstract

**Introduction:** Early graft dysfunction (EAD) complicates liver transplantation (LT). The aim of this analysis was to discriminate between the weight of each variable as for its predictive value toward patient and graft survival.

**Methods:** We reviewed all LT performed at the Medical University of Innsbruck between 2007 and 2018. EAD was recorded when one of the following criteria was present: (i) aspartate aminotransferase (AST) levels >2,000 IU/L within the first 7 days, (ii) bilirubin levels ≥10mg/dL or (iii) international normalized ratio (INR) ≥1.6 on postoperative day 7.

**Results:** Of 616 LT, 30.7% developed EAD. Patient survival did not differ significantly (*P* = 0.092; log rank-test = 2.87), graft survival was significantly higher in non-EAD patients (*P* = 0.008; log rank-test = 7.13). Bilirubin and INR on postoperative day 7 were identified as strong mortality predictors (Bilirubin HR = 1.71 [1.34, 2.16]; INR HR = 2.69 [0.51, 14.31]), in contrast to AST (HR = 0.91 [0.75, 1.10]). Similar results were achieved for graft loss estimation. A comparison with the Model for Early Allograft Function (MEAF) and the Liver Graft Assessment Following Transplantation (L-GrAFT) score identified a superior discrimination potential but lower specificity.

**Conclusion:** Contrarily to AST, bilirubin and INR have strong predictive capacity for patient and graft survival. This fits well with the understanding, that bile duct injury and deprivation of synthetic function rather than hepatocyte injury are key factors in LT.

## Introduction

Liver transplantation (LT) is the treatment of choice for patients with liver failure and select cancer (1–4). Because of a consistent shortage of available grafts, strategies to extend the donor pool such as using organs from expanded-criteria donors or donation after cardiac death (DCD) have evolved (5). The predictive value of organ-quality assessment before transplantation is limited, hence early predictors for the eventual outcome have received attention (6, 7).

To evaluate post-operative organ quality and function, liver function parameters, histology, as well as less invasive procedures were established (8). Primary graft dysfunction can be subdivided into early graft dysfunction (EAD) and primary non-function (9). Several studies aimed to establish a valid definition of EAD and to demonstrate the predictive value (9). Recipients developing EAD experience a longer intensive care unit and hospital stay and have increased mortality and graft loss rates (10). EAD correlates with donor and recipient characteristics, but also with the transplant procedure (11). The most widely used definition of EAD was introduced by Olthoff et al. working from 300 LT recipients at three different sites in the United States and includes (i) bilirubin ≥ 10 mg/dL on postoperative day 7; (ii) international normalized ratio (INR) ≥ 1.6 on postoperative day 7; (iii) alanine aminotransferase (ALT) or aspartate aminotransferase (AST) ≥2,000 IU/mL within the first 7 days (12). Since this definition of EAD is used as an endpoint in clinical and translational studies, it is important to determine the predictive value for the eventual outcome. Primary graft dysfunction as defined by Olthoff et al., occurs in 5.2%−38.7 of liver transplants (11, 12). The relevance of a binary categorization of patients on the long-term outcome and the weighting and interpretation of the individual parameters is lacking. This issue has recently attracted attention, since the introduction of novel techniques to improve organ preservation such as machine perfusion (5, 13), but also the increasing use of livers from DCD donors require early clinical endpoints for assessment of the benefit. Hence, the relevance of AST peak as an important clinical endpoint needs to be reconsidered (9, 14, 15). AST reflects hepatocyte damage but may be of limited value in predicting bile duct injuries. Bile duct injuries, however, have emerged as the most relevant factor determining the fate of an organ in the DCD era (16, 17). A more recent assessment identified lactate, bilirubin and synthesis of coagulation factors as most sensitive clinical predictors for graft dysfunction (9). Despite the large body of literature on EAD, analyses are mainly based on data with short-term follow-up. Organ failure resulting from cholangiopathies may not be captured in a 30- or 90-day follow up period and may be missed in these assessments (9). Moreover, significant knowledge regarding EAD is originated in the early LT years prior to the current model for end-stage liver disease (MELD) era. Thus, we hypothesize that the relevance and significance of EAD has changed over the years and that a reflection on the parameters defining EAD as well as a comparison with other, emerging assessment tools is desired.

We herein aspire to discriminate the individual effect of each variable on the clinical short and long-term outcome in LT. Furthermore, we aim for the identification of potential risk factors for EAD in a European transplant center.

## Methods

This study was conducted in compliance with the Declaration of Helsinki and with local and national regulations. The study protocol was approved by the Internal Medical Review Board (protocol-number EK 1077/2018). The reporting of this study conforms with the STROBE guidelines (18). All primary LT performed between January 1st, 2007 and December 31st, 2017 at the Medical University of Innsbruck (Austria) were reviewed retrospectively. Exclusion criteria were age <18 years, living donor transplantation and split grafts. Donor, recipient and surgical data were obtained from an internal database. The study population was divided into two groups (EAD and non-EAD). According to the definition by Olthoff et al., EAD was recorded when at least one of the following criteria was present: (i) AST levels >2,000 IU/L within the first 7 days post-transplant, (ii) bilirubin levels ≥10mg/dL on postoperative day 7, (iii) INR ≥1.6 on postoperative day 7 (12). Additionally, in order to evaluate the impact of more recently validated kinetic scores, allowing a more accurate, individualized survival risk estimation, the predictive value of the Model for Early Allograft Function (MEAF) score (14) and the Liver Graft Assessment Following Transplantation (L-GrAFT) score (9, 19) were compared to the binary less accurate EAD definition in our cohort.

The following donor data were collected: donation after brain or cardiocirculatory death (DBD/DCD), age, sex, donor risk index (DRI), body mass index (BMI), blood type, cause of death, length of intensive care unit stay, vasopressor use, steatosis in time-zero biopsy, allocation, arterial anatomy, serum parameters. Surgical procedure data included cold ischemia time (CIT), anastomosis time, simultaneous transplantation of more organs, preservation solution, anhepatic phase, duration of surgery, arterial lactate and surgical experience. Recipient data comprised age, sex, BMI, blood type, indication for LT, hepatitis C virus (HCV) status, biological MELD score, Child Pugh Score and Surgical Risk Score at LT. Following post-operative factors for clinical outcomes and complications were achieved: length of hospital stay, Clavien Dindo post-operative complication rate, transplant-related reoperations, vascular and biliary complications, graft and patient survival. Transplant-related reoperations were classified as re-interventions within 30 days of the first surgery, including surgeries for intra-abdominal bleeding or fluid collection and treatment of vascular or biliary complications. Length of hospital stay was defined as the time from LT to hospital discharge. Vascular complications included hepatic artery thrombosis, stenosis, or dissection, portal vein stenosis, portal or supra-hepatic venous thrombosis. Biliary complications included bile duct leakage, anastomotic and non-anastomotic strictures.

### Statistical Analysis

Data were presented as proportions (%), means ± standard deviation (SD) or medians as appropriate. Factors associated with EAD were investigated using logistic regression analysis. Results were expressed as estimated odds ratio (OR), 95% confidence interval (CI) and *P*-value. Comparative analysis of clinical outcomes of patients in the EAD and non-EAD group was conducted using the Chi-square and Fisher's Exact Test for categorical variables and Mann-Whitney-U-Wilcoxon Test for continuous variables. Mann-Whitney-Wilcoxon tests are approximated by using the *t* or *F* distributions. Patient and graft survival were evaluated at different time points: 1, 3, 5 years. Graft survival was calculated from the transplant date until the date of re-transplantation or death (whichever came first). The discriminant ability of individual components of EAD definition was assessed by developing a Cox proportional hazard model using the Wald test with backwards stepwise selection. Results were expressed as Hazard Ratio (HR 95%CI) and *P*-value. All information outside the periods of interest was censored for the date of the end of the study period or for the date of the last correspondence. Patient and graft survival rates were compared using the Kaplan–Meier method. The log-rank test was applied for comparative analysis of EAD and non-EAD groups. For descriptive analysis and survival curves, two tailed *P*-values <0.05 after adjustments for confounding were considered significant throughout the analysis. Discrimination (ability of the model to accurately classify patients in predicting patient and graft failure free survival) was assessed by computing the area under the receiver operator characteristic curve (AUROC) and its 95% confidence intervals. The AUROC and the differences in AUROCs were estimated across the bootstrapped imputed datasets and averaged. The 95% confidence intervals (CI) for the point estimates were computed using the percentile method. Sensitivity analyses were performed using complete cases where not a single data point was missing and the results compared to the imputed analyses. Statistical analyses were performed using *R* statistical software (Team RC, R Foundation for Statistical Computing, Vienna, Austria) (20).

## Results

**Figure 1:**
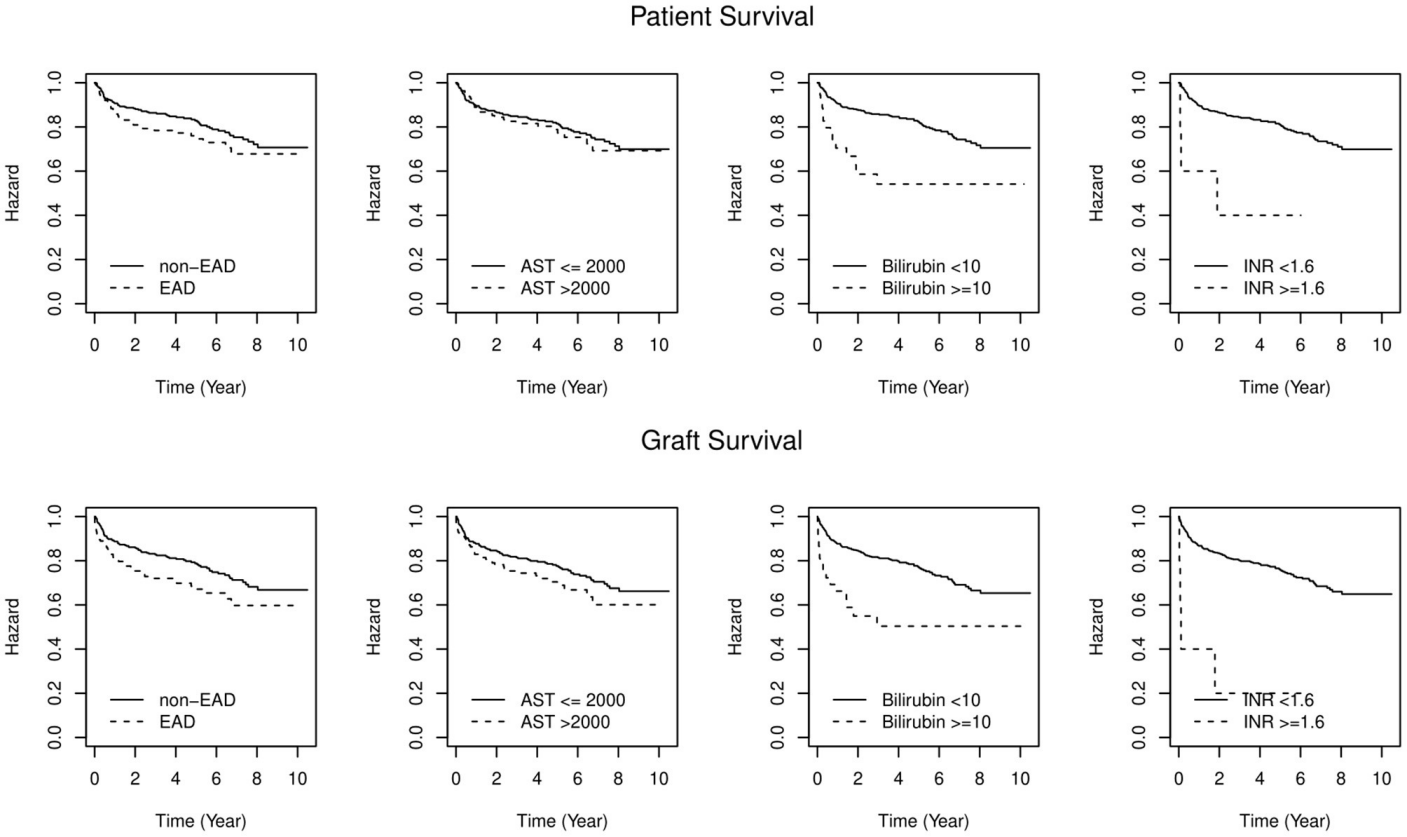
Patient and Graft survival by EAD status. Difference in overall patient and graft survival between EAD and non-EAD groups (*P* = 0.092; log rank-test = 2.87; *P* = 0.008; log rank-test = 7.13). Levels of significance: *P*-values <0.05. EAD, Early allograft dysfunction; AST, Aspartate aminotransferase; INR, International normalized ratio.

Between January 2007 and December 2017, 616 LT with allografts from DCD and DBD donors were performed at the Medical University of Innsbruck. Recipient aged <18 years, living donors and split liver grafts were excluded from the analysis. Hundred-eighty-nine recipients (30.7%) developed EAD. This rate varied between 12 and 21% in the early years (2007–2009), while in the following years (2010–2018) a trend toward higher rates (between 32 and 40%) was observed. The 1-, 3-, and 5-year patient survival rates were 91/86/83% vs. 86/78/76% in patients without EAD vs. those with EAD (W = 2.84, *P* = 0.092) respectively. Allograft survival rates in the non-EAD group vs. the EAD group at 1, 3, and 5 years were 89/83/79% vs. 81/72/67%, respectively (*W* = 7, *P* = 0.008; Figure 1; Table 1). Similar results were observed concerning patient and graft survival after 1 year (Supplementary Figure 1). The contribution of individual components to the diagnosis of EAD in reference to mortality and graft loss are displayed in Tables 1, 2. Isolated elevated AST (78%) was mostly determining EAD in our cohort, followed by bilirubin (8%). A minority of recipients with EAD met more than one variable requirement (13%). AST levels eventually returned to normal in all patients at 1 year. Bilirubin values also decreased to normal levels in the majority of patients at 1 year (6% vs. 1% and 18% vs. 1%). Contrarily, INR values remained constantly elevated (1% vs. 3% vs. 2% vs. 2%), respectively.

**Table 1:** Patient and graft survival in years.

**Patient**		***n*** **(% [95% CI])**			**Wald-test, *P*-value**
	***N***	**1**	**3**	**5**	
EAD	189	23 [81%, 92%]	11 [72%, 85%]	2 [69%, 83%]	*W* = 2.84, *p* = 0.092
Non-EAD	427	36 [88%, 94%]	14 [83%, 90%]	8 [79%, 87%]	
AST ≤ 2,000	444	42 [87%, 93%]	16 [81%, 89%]	8 [77%, 86%]	*W* = 0.31, *p* = 0.580
AST 2,000	172	17 [84%, 94%]	9 [75%, 88%]	2 [72%, 87%]	
Bilirubin <10	578	49 [88%, 93%]	21 [83%, 89%]	10 [79%, 86%]	*W* = 12.64, *p* < 0.001
Bilirubin ≥ 10	38	10 [57%, 88%]	4 [38%, 76%]	0 [38%, 76%]	
INR ≤ 1.6	611	57 [87%, 92%]	24 [81%, 88%]	10 [78%, 85%]	*W* = 6.82, *p* = 0.009
INR ≥ 1.6	5	2 [29%, 100%]	1 [14%, 100%]	0 [14%, 100%]	
MEAF ≤ 5	260	22 [87%, 94%]	10 [81%, 91%]	4 [78%, 89%]	*W* = 4.32, *p* = 0.038
MEAF ≥ 7	87	9 [82%, 96%]	6 [70%, 89%]	2 [64%, 86%]	
MEAF = [5,7]	269	28 [84%, 92%]	9 [79%, 89%]	4 [74%, 86%]	
**Graft**	**N**	**1**	**3**	**5**	**Wald-test**, ***P*****-value**
EAD	189	33 [75%, 87%]	12 [65%, 79%]	4 [60%, 76%]	*W* = 7.00, *p* = 0.008
Non-EAD	427	44 [86%, 92%]	19 [79%, 87%]	9 [74%, 83%]	
AST ≤ 2,000	444	50 [85%, 91%]	21 [78%, 85%]	9 [73%, 82%]	*W* = 3.19, *p* = 0.074
AST ≥ 2,000	172	27 [77%, 89%]	10 [67%, 82%]	4 [61%, 78%]	
Bilirubin <10	578	65 [85%, 91%]	27 [78%, 85%]	13 [73%, 81%]	*W* = 13.17, *p* < 0.001
Bilirubin ≥ 10	38	12 [52%, 84%]	4 [35%, 72%]	0 [35%, 72%]	
INR ≤ 1.6	611	74 [84%, 90%]	30 [77%, 84%]	13 [72%, 80%]	*W* = 14.97, *p* < 0.001
INR ≥ 1.6	5	3 [14%, 100%]	1 [3%, 100%]	0 [3%, 100%]	
L-GrAFT < −2	55	8 [74%, 95%]	3 [64%, 90%]	0 [64%, 90%]	*W* = 54.55, *p* < 0.001
L-GrAFT > +2	27	12 [38%, 77%]	3 [18%, 65%]	0 [18%, 65%]	
L-GrAFT = [−2, +2]	534	57 [85%, 91%]	25 [78%, 86%]	13 [73%, 82%]	
MEAF ≤ 5	260	26 [85%, 93%]	13 [78%, 88%]	5 [74%, 85%]	*W* = 11.39, *p* < 0.001
MEAF ≥ 7	87	16 [72%, 89%]	7 [58%, 81%]	2 [52%, 77%]	
MEAF = [5,7]	269	35 [81%, 90%]	11 [75%, 85%]	6 [68%, 81%]	

*Proportional hazards regression model*.

*EAD, Early allograft dysfunction; AST, Aspartate aminotransferase; INR, International normalized ratio; MEAF, Model for Early Allograft Function; L-Graft, Liver Graft Assessment Following Transplantation*.

**Table 2:** Early allograft dysfunction univariate analysis for EAD risk factors.

**Item**	**Non-EAD**	**EAD**	***P*-value**
**Recipient characteristics**			
Recipient age (Years) (median)	58 (IQR 14)	59 (IQR 10)	*p* = 0.074
Body mass index (kg/m^2^) (median)	25.10 (IQR 5.80)	25.90 (IQR 6.00)	*p* = 0.034
Recipient sex			*p* = 0.030
Male	75% (321)	83% (157)	
Female	25% (106)	17% (32)	
MELD Score (mean)	21.11 (SD 9.06)	22.08 (SD 9.42)	*p* = 0.238
CHILD Score			*p* < 0.001
A	18% (76)	32% (61)	
B	54% (230)	41% (78)	
C	28% (121)	26% (50)	
AB0			*p* = 0.795
A	41% (173)	45% (84)	
B	11% (46)	10% (18)	
0	41% (173)	40% (74)	
AB	6% (25)	5% (9)	
Hepatitis C			*p* = 0.003
Negative	91% (387)	97% (184)	
Positive	9% (40)	3% (5)	
Re-transplantation	4% (18)	11% (21)	*p* = 0.001
Acute liver failure	4% (15)	5% (9)	*p* = 0.460
Encephalopathy	23% (97)	20% (37)	*p* = 0.384
Cirrhosis	95% (407)	93% (176)	*p* = 0.265
Tumor	30% (127)	42% (80)	*p* = 0.002
Hepatocellular carcinoma	29% (123)	42% (79)	*p* = 0.002
Tumor entity			*p* = 0.029
No tumor	70% (301)	58% (109)	
Hepatocellular carcinoma	28% (120)	41% (78)	
Cholangiocellular• carcinoma	<1% (3)	<1% (1)	
Neuroendocrine tumor	<1% (1)	.	
Liver metastases of colon• malignancy	.	.	
Other	<1% (2)	<1% (1)	
Tumor treatment	28% (118)	39% (73)	*p* = 0.007
Transarterial chemoembolization	15% (63)	21% (40)	*p* = 0.049
Radiofrequency ablation	14% (61)	21% (40)	*p* = 0.033
Surgical risk score			*p* = 0.746
None	63% (269)	59% (111)	
Low	22% (94)	23% (44)	
Middle	5% (22)	6% (12)	
High	10% (42)	12% (22)	
Portal vein open			*p* = 0.293
Yes	85% (365)	83% (157)	
partial obstruction of portal vein trunk	12% (50)	11% (21)	
complete obstruction of portal vein trunk	2% (9)	4% (7)	
thrombosis right or left portal branch	<1% (3)	2% (4)	
**Donor characteristics**			
Donor age (years) (median)	51 (IQR 25)	53 (IQR 19)	*p* = 0.163
Donor sex			*p* < 0.001
Male	52% (223)	68% (129)	
Female	48% (204)	32% (60)	
Body mass index (kg/m^2^) (median)	25.00 (IQR 4.80)	27.00 (IQR 4.30)	*p* < 0.001
Donor height (cm) (mean)	172.99 (SD 8.82)	175.68 (SD 8.52)	*p* < 0.001
Donor weight (kg) (mean)	75.97 (SD 13.96)	85.58 (SD 14.39)	*p* < 0.001
Steatosis			*p* < 0.001
None	63% (267)	38% (71)	
Mild	32% (133)	37% (70)	
Medium	5% (21)	20% (38)	
Severe	.	5% (10)	
Donor risk index (mean)	1.80 (SD 0.34)	1.93 (SD 0.51)	*p* = 0.001
Donor artery			*p* = 0.286
Normal anatomy	81% (343)	84% (159)	
Variance	19% (83)	16% (30)	
Allocation			*p* = 0.015
Local	43% (184)	34% (65)	
Regional	37% (159)	50% (94)	
National	20% (84)	16% (30)	
Cause of death			*p* = 0.819
Trauma	24% (104)	22% (42)	
Anoxia	11% (48)	10% (19)	
Cardiovascular accident	63% (267)	65% (123)	
Other	2% (8)	3% (5)	
Sodium (U/L) (mean)	146.50 (SD 12.87)	147.74 (SD 7.81)	*p* = 0.579
Preservation solution			*p* = 0.843
University of Wisconsin	23% (98)	23% (43)	
Histidine-tryptophan-•ketoglutarate	75% (320)	74% (140)	
Other	2% (9)	3% (6)	
**Operative data**			
Simultaneous transplant			*p* = 0.185
No	94% (401)	97% (184)	
Liver–kidney	6% (25)	3% (5)	
Heart–liver	.	.	
Multi-organ transplantation	<1% (1)	.	
Anhepatic phase (min) (mean)	52.89 (SD 11.91)	58.14 (SD 13.92)	*p* < 0.001
Surgery duration (min) (mean)	395.57 (SD 108.22)	433.40 (SD 123.14)	*p* < 0.001
Mass clamping ligamentum hepatoduodenale	<1% (2)	1% (2)	*p* = 0.401
Anastomosis time (min) (mean)	44.49 (SD 10.78)	52.61 (SD 25.35)	*p* < 0.001
Cold Ischemia time (h) (mean)	8.55 (SD 2.50)	9.13 (SD 2.29)	*p* = 0.003
Arterial lactate (mean)	24.74 (SD 21.59)	34.99 (SD 32.64)	*p* < 0.001
Surgical experience			*p* = 0.145
<50 liver transplantations	35% (148)	58% (109)	
≥50 liver transplantations	65% (279)	42% (80)	

*Chi-square and Fisher's Exact Test for categorical variables; Mann-Whitney-U-Wilcoxon Test for continuous variables*.

*EAD, Early allograft dysfunction; MELD, Model for end-stage liver disease*.

**Figure 2:**
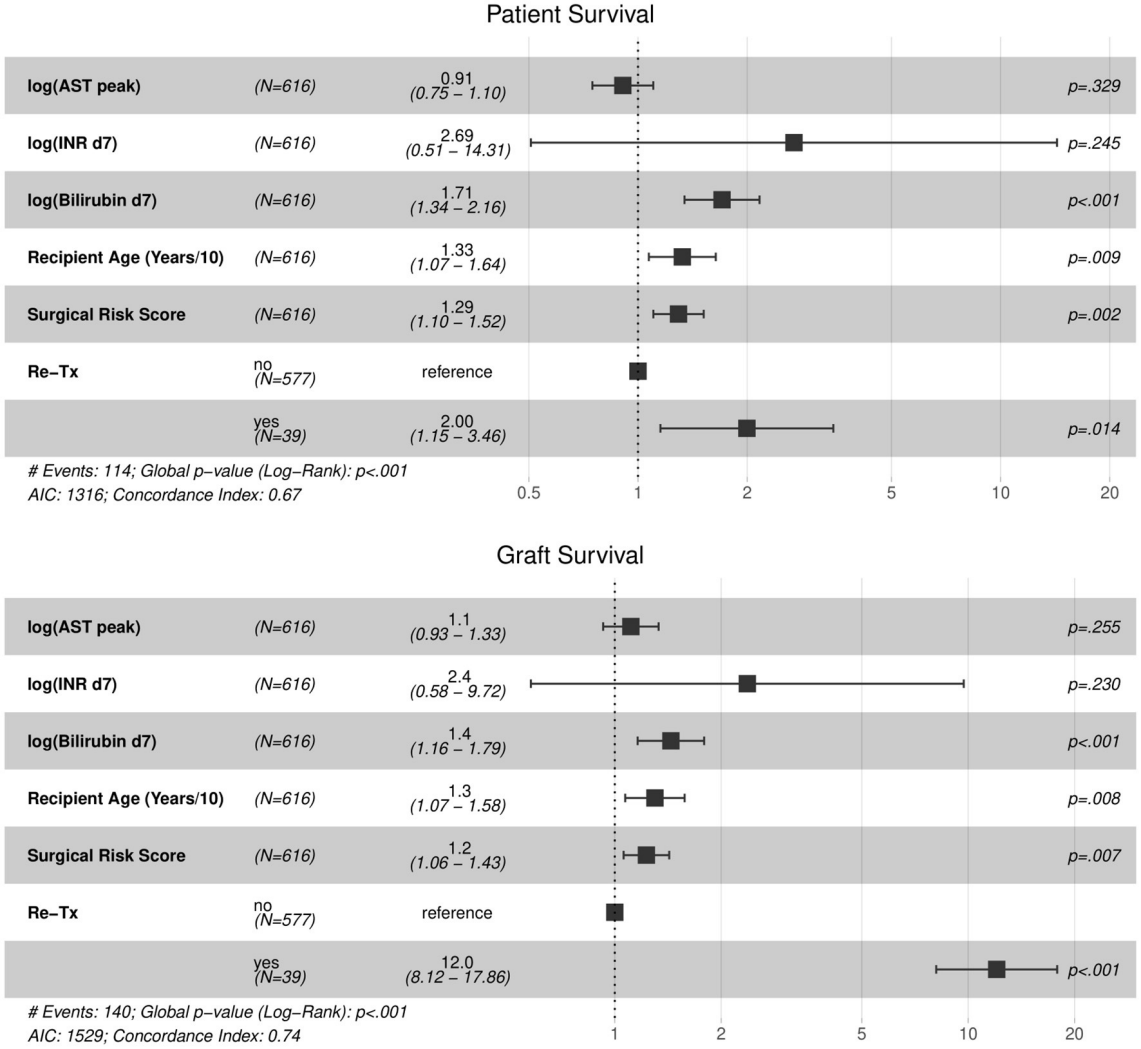
Patient and Graft Cox proportional regression model. Comparison of Hazard Ratios (HR) for patient survival: bilirubin HR = 1.71; INR HR = 2.69, AST HR = 0.91. Comparison of HR for graft survival: bilirubin HR = 1.44; INR HR = 2.37; AST HR = 1.11. Levels of significance: *P*-values <0.05. EAD, Early allograft dysfunction; AST, Aspartate aminotransferase; INR, International normalized ratio; HR: Hazard Ratio.

Survival rates and risk factors are shown in Table 1. EAD-AST patients did not display a higher mortality risk compared to non-EAD patients. In contrast, patients who showed an elevated INR (EAD-INR) and total bilirubin (EAD-BILIRUBIN) on day 7 had a significantly inferior patient and graft survival. The proportional hazard regression model allowed to assess single variables for discrimination of 1-, 3-, and 5-year mortality. While bilirubin was confirmed as the best predictor of mortality (*W* = 12.64, *P* < 0.001 for patient survival; *W* = 13.17, *P* < 0.001 for graft survival), AST did not serve to display a significant discrimination (*W* = 0.31, *P* = 0.580 for patient survival; *W* = 3.19, *P* = 0.074 for graft survival, Figure 1). As additional information to estimate the probability of mortality and graft loss, a Cox proportional hazard model was designed (Figure 2). Comparing the Hazard Ratio (HR) of each variable, bilirubin and INR were selected as strong mortality predictors.

(Bilirubin HR = 1.71 [1.34, 2.16]; INR HR = 2.69 [0.51, 14.31]), while AST did not reach significant effect (HR = 0.91 [0.75, 1.10]) suggesting a categorized discrimination potential of individual variables in EAD status. Similar results were achieved for graft loss estimation (Bilirubin HR = 1.44 [1.16, 1.79]; INR HR = 2.37 [0.58, 9.72]; AST HR = 1.11 [0.93, 1.33] (Figure 2; Supplementary Table 1).

As a next step, the predictive value of the MEAF score and the L-GrAFT score were compared to the binary EAD definition in our cohort. The L-GrAFT model had a *C* statistic of 0.629 with a superior discrimination of graft survival compared with the existing EAD definition (*C* statistic: 0.542, *P* < 0.001) and the MEAF score (*C* statistic: 0.551, *P* < 0.001), respectively. Concerning patient survival, the MEAF score showed a superior discrimination potential compared to the binary EAD definition (*C* statistic 0.528 vs. 0.527, *P* = 0.038). Both scores revealed a low specificity for patient and graft survival indicating a limited capacity in correctly classifying outcomes in this cohort (Table 1; Supplementary Figure 2; Supplementary Table 2).

**Figure 3:**
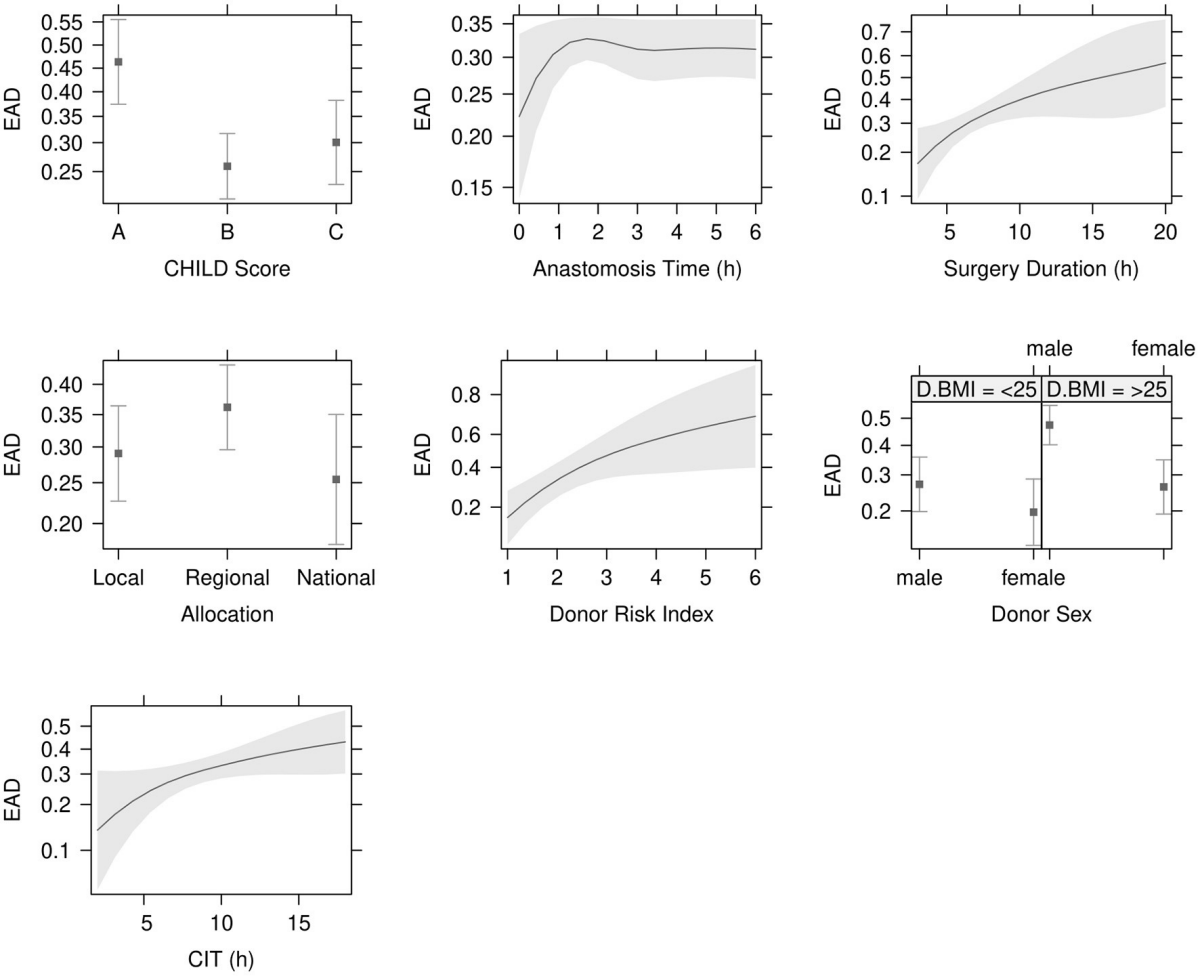
Independent risk factors for EAD. Child *B* Score (*P* < 0.001), Child *C* Score (*P* = 0.007) and surgery duration (*P* = 0.010) were significant recipient and operative donor risk factors in the logistic regression analysis. Cold ischemia time (CIT) (*P* < 0.049), donor risk index (DRI) (*P* = 0.009) and donor BMI > 25 (OR = 2.43, 95% CI: 1.49–4.01, *P* < 0.001) were independent donor risk factors. Levels of significance: *P*-values <0.05. EAD: Early allograft dysfunction; BMI: Body mass index.

Data concerning donor characteristics, surgical procedure and recipient demographics are displayed in Table 2. The mortality rate was 19% (114/616) and the re-transplantation rate was 4% in the non-EAD compared to 11% in the EAD group. Clinical outcomes and complications are displayed in Table 3. EAD risk factor analysis is shown in Table 2. The variables with the strongest significance in the single factor regression analysis were included in the multiple regression model (Table 4). Child *B* Score (OR = 0.40, 95% CI: 0.26–0.64, *P* < 0.001), Child *C* Score (OR = 0.50, 95% CI: 0.30–0.82, *P* = 0.007), surgery duration (OR = 2.67, 95% CI: 1.27–5.63, *P* = 0.01) CIT (OR = 2.04, 95% CI: 1.01–4.19, *P* < 0.049), donor risk index (DRI) (OR = 4.02, 95% CI: 1.42–11.61, *P* = 0.009) and donor BMI > 25 (OR = 2.43, 95% CI: 1.49–4.01, *P* < 0.001) were identified as independent recipient-, surgery- and donor-related risk factors (Figure 3 and Table 4).

**Table 3:** Clinical outcomes and complications.

**Item**	**Non-EAD**	**EAD**	***P*-value**
Intensive care unit length of stay (days) (mean)	5.28 (SD 4.40)	6.14 (SD 4.69)	*p* = 0.007
Length of stay (days) (mean)	24.91 (SD 17.49)	29.52 (SD 22.85)	*p* = 0.006
Clavien Dindo classification			*p* = 0.087
I	2% (9)	3% (6)	
II	15% (64)	17% (32)	
IIIa	12% (51)	10% (19)	
IIIb	20% (85)	16% (30)	
Iva	32% (137)	27% (51)	
IVb	5% (21)	8% (15)	
*V*	14% (60)	19% (36)	
Arterial complications	9% (38)	12% (22)	*p* = 0.271
Arterial dissection	2% (8)	2% (4)	*p* = 0.827
Arterial thrombosis	2% (9)	4% (7)	*p* = 0.242
Arterial stenosis	5% (21)	6% (11)	*p* = 0.625
Portal vein stenosis	2% (7)	4% (7)	*p* = 0.107
Venous thrombosis	3% (12)	4% (8)	*p* = 0.351
Re-operation rate	33% (140)	50% (94)	*p* < 0.001
Re-operation <30 days	27% (116)	42% (78)	*p* < 0.001
Bleeding	13% (55)	23% (43)	*p* = 0.002
Acute kidney failure	4% (15)	5% (9)	*p* = 0.460
Bile duct leakage	14% (58)	22% (41)	*p* = 0.010
Bile duct non-anastomotic stricture	11% (47)	16% (29)	*p* = 0.122
Bile duct anastomotic stricture	28% (121)	26% (49)	*p* = 0.575

*Chi-square and Fisher's exact test for categorical variables; Mann-Whitney-U-Wilcoxon test for continuous variables*.

*EAD, Early allograft dysfunction*.

**Table 4:** Logistic regression analysis for EAD risk factors.

**Term**	***B***	**95%-CI**	***T***	**OR**	**OR 95%-CI**	***P*-value**
CHILD *B* score	−0.906	[−0.453, −1.36]	−3.91	0.40	[0.26, 0.64]	<0.001
CHILD *C* score	−0.698	[−0.194, −1.21]	−2.70	0.50	[0.30, 0.82]	0.007
Anastomosis time (h)	0.458	[1.08, −0.127]	1.49	1.58	[0.88, 2.95]	0.135
Surgery duration (h)	0.981	[1.73, 0.241]	2.59	2.67	[1.27, 5.63]	0.010
Allocation regional	0.325	[0.774, −0.121]	1.43	1.38	[0.89, 2.17]	0.153
Allocation national	−0.183	[0.389, −0.767]	−0.62	0.83	[0.46, 1.47]	0.534
Donor risk index	1.39	[2.45, 0.354]	2.60	4.02	[1.42, 11.61]	0.009
Cold ischemia time (h)	0.711	[1.43, 0.0112]	1.97	2.04	[1.01, 4.19]	0.049
Donor sex [female]	−0.417	[0.207, −1.06]	−1.30	0.66	[0.35, 1.23]	0.195
D-BMI >25	0.886	[1.39, 0.397]	3.51	2.43	[1.49, 4.01]	<0.001
Donor sex [female]: donor BMI >25	−0.508	[0.293, −1.31]	−1.25	0.60	[0.27, 1.34]	0.212

*Model: binomial; pseudo r-squared: McFadden = 0.11, r2ML = 0.13, r2CU = 0.19 Wald-Test*.

*EAD, Early allograft dysfunction; BMI, Body mass index*.

Since our assessment indicated, that the clinical implication of EAD is most significant when the criteria bilirubin and INR are driving the condition, a logistic regression was performed for the EAD cohort under the modification to exclude EAD-AST patients. Despite the comparable statistical effect size, the determination of the mathematical significance is limited due to the resulting small EAD group (*n* = 17). However, analyzing this subgroup descriptively, some relevant common aspects stand out: the re-transplantation rate was 18 and 29% of the recipients were classified as surgically high risk. The donor data revealed that 59% had a mild steatosis and that the cause of death was a cardiovascular event in 88%. Compared to patients without EAD-BILIRUBIN/INR, the 1-, 3-, and 5-year patient survival rate was very low (91% vs. 61%; 86% vs. 47% and 83% vs. 0%; *W* = 11.64, *P* < 0.001). Allograft survival rates in the non-EAD group and the EAD-BILIRUBIN plus EAD-INR group at 1, 3, and 5 years were 89% vs. 61%, 83% vs. 48% and 79% vs. 0%, respectively (*W* = 8.71, *P* = 0.003). Re-operation rate was 76% and amount of biliary complications in the first 30 days reached 41%.

## Discussion

While EAD is a multifactorial clinical condition which correlates with patient and graft outcome, the binary readout does not well specify the type and severity of graft dysfunction since it does not differentiate single parameters (14). In the currently most widely used definition of EAD (12) the combination of parameters for cell/hepatocyte damage, synthetic liver function and bilirubin conversion and excretion are combined into a single endpoint. The considered variables play different roles for liver metabolism, suggesting distinct influence on survival. While this composite parameter summarized the core liver functions, it fails to discriminate the individual parameters according to their clinical impact (9, 19, 21).

Evolving trends suggest different interpretation of single liver function tests. In this setting, machine perfusion is recognized as one of the most significant improvements in the field of transplantation over the past 20 years (15, 22). This development and the need for reliable and robust endpoints for clinical trials puts further emphasis on the necessity to refine the definition for EAD. Recently, Pareja et al., (14) introduced the MEAF score, which allows a continuous grading of EAD, but does not assess the models' accuracy in reference to graft failure. Agopian et al., developed the L-GrAFT score (9) for the individualized calculation of graft failure risk following LT and compared its prognostic performance with the binary EAD definition and MEAF score. The L-GrAFT risk score model allows for highly accurate, individualized risk estimation of 3-month graft failure following LT and is superior to the existing binary EAD classification and MEAF score. Further to this, it was recently validated in a multicenter analysis (19). However, the mathematically more complex L-GrAFT score requires data from the first 10 days post-LT and may be cumbersome.

We observed an EAD incidence of 30.7% in our cohort. This is in line with other reports identifying EAD rates ranging from 5.2 to 38.7% (11, 12). Parenchymal injury as measured by elevated transaminases alone (EAD-AST), did not have a significant impact on patient and graft survival. This indicated that hepatic damage with little involvement of the biliary tree may cause liver tissue damage, but that the clinical manifestation has a more benign phenotype when compared to conditions that possibly cause cholangiopathy (23). When hepatocyte injury occurs, the transaminase level, especially AST, increases in the serum (24). The liver has a regenerative capacity and can tolerate significant loss of hepatocytes. Accordingly, a normalization of AST levels was found in all EAD patients after 1 year. The relation between EAD and the post-operative outcome after LT demonstrated that EAD patients had a higher rate of vascular and bile duct complications, more frequent transplant-related reoperations and a prolonged hospital stay. Only INR and bilirubin contribute to an inferior patient survival, confirming the relevance and predictive capacity of these parameters toward hard endpoints. Hence, the weight of different parameters in the definition of EAD should be considered. The analysis of the patients fulfilling the EAD criteria based on bilirubin and INR revealed a detrimental effect of these two parameters with respect to the biliary complications, long-term patient and graft survival rates.

In line with other analyses (12, 25), the majority of patients in our cohort met the AST criterion. However, when assessing AST as a single factor, the sub-analysis showed similar patient and graft survival rates implying a less relevant role of transaminases regarding the outcome. Patients with elevated bilirubin or INR displayed a significantly different patient and graft survival. Biliary complications were mostly found in patients matching the bilirubin criterion. Thus, parenchymal damage seems to be overrated as a biomarker since the fate of the organ is more often determined by biliary tree injuries (23). Learning from the clinical application of new technologies like machine perfusion, it seems that only substantial and/or progressive hepatocellular damage is clinically relevant. The intraoperative assessment of arterial lactate concentration at the end of LT is an early biomarker for EAD (26). This was confirmed in our cohort where arterial lactate was significantly elevated in EAD patients compared to non-EAD patients (34.99 vs. 24.74; *P* < 0.001).

The association of EAD with donor risk factors, recipient characteristics and intra-operative events revealed an association between BMI > 25 kg/m^2^ and EAD. Recipient sex is weakly associated with transplant outcomes. Sex may be indirectly related to graft size and liver mass since the trend relates to male recipient with BMI > 25 kg/m^2^ displaying a higher rate of EAD. Since graft steatosis also correlates with EAD, we hypothesize that increased hepatocyte loss in larger steatotic livers is a contributing factor to this finding. Future analyses might consider to adjust for donor and recipient liver mass to accurately identify EAD (8). EAD is linked to ischemia/reperfusion (I/R) injury after transplantation (27). I/R injuries were mostly related to DCD and graft steatosis (28). A recent meta-analysis concluded that livers with mild steatosis are robust liver grafts, while moderately and severely steatotic livers require short CIT times for good outcomes (29). Although histopathological I/R injury assessment was not analyzed in our study, moderate and severe graft steatosis was found to play a significant role with an increment to the relative risk for EAD of 25–30%.

The DRI introduced by Feng et al., is based on selected donor characteristics and permits quantitative graft dysfunction risk assessment (30, 31). The predictive value was confirmed in our study. The importance of surgery duration and ischemia time was often highlighted in the early and late post-transplant outcomes. Prolonged CIT ≥10 h and anastomosis time ≥40 min were associated with EAD (8). Our results confirmed prolonged ischemia time as potentially relevant risk factor for EAD. In this study, local donors accounted for 43% of the non-EAD and 34% of the EAD, while regional donors corresponded with 37% of non-EAD and 50% of EAD patients. One aspect explaining these findings might be related to different center-specific policies in coordinating the donor and recipient equips, in order to keep the ischemic time as short as possible. Moreover, patients who undergo a more complex hepatectomy as a result of the anatomical condition or portal hypertension are more likely to have prolonged ischemia times. Duration of surgery was found as independent risk factor for EAD in this analysis. In order to evaluate the influence of surgical experience on duration of surgery and outcome, an auxiliary differentiation between surgeons who performed more than 50 LT vs. <50 LT was added. This assessment did not show significant differences (*p* = 0.145).

The strength of the analysis is the relatively large and well documented cohort, the overall uniform practice within our department regarding standardized surgical techniques, medical treatment and postoperative management. However, the retrospective study design should be considered as limitation. Future multi-center studies are warranted to confirm our results.

In summary, this single center study provides new insights into the utility of EAD as end-point in LT. Our findings demonstrated a diverse impact of the single parameters contributing to the EAD definition, indicating different clinical conditions and possibly requiring different interventions. While EAD was associated with inferior graft survival, only bilirubin and INR weighted in on this correlation. Individual discrimination of each variable provides more precise information than current categorical classifications. This analysis displayed a comparable patient survival, probably related to the attenuation of the currently cited risk factors for EAD during the last decades. Eventually, the redefinition of EAD as an early endpoint with predictive value for patient and graft survival is necessary to enable further advancement in LT (15). Pre-existing endpoints such as EAD are gradually integrated by novel and combined endpoints, or surrogate endpoints, still requiring further confirmation before routine use. Since innovation in LT is thriving again, the accurate validation of robust endpoints in consideration of all stakeholders is of key importance and demands a joining of forces, involvement of patients' viewpoints and close interaction with the regulatory bodies.

## Data Availability Statement

The original contributions presented in the study are included in the article/Supplementary Material, further inquiries can be directed to the corresponding author/s.

## Ethics Statement

The studies involving human participants were reviewed and approved by the Innsbruck Medical University Internal Medical Review Board (Protocol Number EK 1077/2018). The patients/participants provided their written informed consent to participate in this study.

## Author Contributions

MF, WP, and SS: conceptualization and validation. MF, AWo, AW, HE, and WP: data curation. MF, AWo, WP, and SS: formal analysis. MF and SS: investigation, project administration, resources, writing—original draft. MF, AWo, and WP: methodology. MF and WP: software. SS: supervision. WP, HE, RO, CM, MM, BC, TR, AWe, RS, HZ, HT, DÖ, and SS: visualization. MF, WP, HE, RO, CM, MM, BC, TR, AWe, RS, HZ, HT, DÖ, and SS: writing—review and editing. All authors contributed to the article and approved the submitted version.

## Funding

This work was supported by Dr. Gabriel Salzner Stiftung.

## Conflict of Interest

The authors declare that the research was conducted in the absence of any commercial or financial relationships that could be construed as a potential conflict of interest.

## Publisher's Note

All claims expressed in this article are solely those of the authors and do not necessarily represent those of their affiliated organizations, or those of the publisher, the editors and the reviewers. Any product that may be evaluated in this article, or claim that may be made by its manufacturer, is not guaranteed or endorsed by the publisher.

## Abbreviations

ALT: Alanine aminotransferase
AST: Aspartate aminotransferase
BMI: Body mass index
CIT: Cold ischemia time
DCD: Donors after cardiac death
DBD: Donors after brain death
DRI: Donor risk index
EAD: Early graft dysfunction
INR: International normalized ratio
L-GrAFT: Liver Graft Assessment Following Transplantation
LT: Liver transplantation
MEAF: Model for Early Allograft Function
MELD: Model for end-stage liver disease.

## References

[1] Saidi RF. Utilization of expanded criteria donors in liver transplantation. Int J Organ Transplant Med. (2013) 4:46–59. 25013654 PMC4089311

[2] Saidi RF. Current status of liver transplantation. Arch Iran Med. (2012) 15:772–6. 10.0121512/AIM.0011 23199251

[3] Adam R Karam V Cailliez V Grady JGO Mirza D Cherqui D. 2018 Annual Report of the European Liver Transplant Registry (ELTR)−50-year evolution of liver transplantation. Transpl Int. (2018) 31:1293–317. 10.1111/tri.13358 30259574

[4] Pfitzmann R Nüssler NC Hippler-Benscheidt M Neuhaus R Neuhaus P. Long-term results after liver transplantation. Transpl Int. (2008) 21:234–46. 10.1111/j.1432-2277.2007.00596.x 18031464

[5] Schlegel A Dutkowski P. Role of hypothermic machine perfusion in liver transplantation. Transpl Int. (2005) 28:677–89. 10.1111/tri.12354 24852621

[6] Cardini B Fodor M Hermann M Wieser V Hautz T Mellitzer V. Live confocal imaging as a novel tool to assess liver quality: insights from a murine model. Transplantation. (2020) 104:2528–37. 10.1097/TP.0000000000003405 33215899

[7] Cardini B Oberhuber R Fodor M Hautz T Margreiter C Resch T. Clinical implementation of prolonged liver preservation and monitoring through normothermic machine perfusion in liver transplantation. Transplantation. (2020) 104:1917–28. 10.1097/TP.0000000000003296 32371845

[8] Bastos-Neves D Salvalaggio PRO Almeida MD. Risk factors, surgical complications and graft survival in liver transplant recipients with early allograft dysfunction. Hepatobiliary Pancreat Dis Int. (2019) 18:423–9. 10.1016/j.hbpd.2019.02.005 30853253

[9] Agopian VG Harlander-Locke MP Markovic D Dumronggittigule W Xia V Kaldas FM. Evaluation of early allograft function using the liver graft assessment following transplantation risk score model. JAMA Surg. (2018) 153:436–44. 10.1001/jamasurg.2017.5040 29261831 PMC6584313

[10] Pokorny H Gruenberger T Soliman T Rockenschaub S Längle F Steininger R. Organ survival after primary dysfunction of liver grafts in clinical orthotopic liver transplantation. Transpl Int. (2000) 13:S154–7. 10.1111/j.1432-2277.2000.tb02009.x 11111986

[11] Neves DB Rusi MB Diaz LG Salvalaggio P. Primary graft dysfunction of the liver: definitions, diagnostic criteria and risk factors. Einstein. (2016) 14:567–72. 10.1590/s1679-45082016rw3585 27783749

[12] Olthoff KM Kulik L Samstein B Kaminski M Abecassis M Emond J. Validation of a current definition of early allograft dysfunction in liver transplant recipients and analysis of risk factors. Liver Transpl. (2010) 16:943–9. 10.1002/lt.22091 20677285

[13] Ravikumar R Leuvenink H Friend PJ. Normothermic liver preservation: a new paradigm? Transpl Int. (2015) 28:690–9. 10.1111/tri.12576 25847684

[14] Pareja E Cortes M Hervás D Mir J Valdivieso A Castell JV. A score model for the continuous grading of early allograft dysfunction severity. Liver Transpl. (2015) 21:38–46. 10.1002/lt.23990 25204890

[15] Dutkowski P Guarrera JV de Jonge J Martins PN Porte RJ Clavien PA. Evolving trends in machine perfusion for liver transplantation. Gastroenterology. (2019) 156:1542–7. 10.1053/j.gastro.2018.12.037 30660724

[16] Schlegel A Muller X Kalisvaart M Muellhaupt B Perera MTPR Isaac JR. Outcomes of DCD liver transplantation using organs treated by hypothermic oxygenated perfusion before implantation. J Hepatol. (2019) 70:50–7. 10.1016/j.jhep.2018.10.005 30342115

[17] Schlegel A Dutkowski P. Impact of machine perfusion on biliary complications after liver transplantation. Int J Mol Sci. (2018) 19:3567. 10.3390/ijms19113567 30424553 PMC6274934

[18] Cheng A Kessler D Mackinnon R Chang TP Nadkarni VM Hunt EA. International Network for Simulation-based Pediatric Innovation, and Education (INSPIRE) reporting guidelines investigators: reporting guidelines for health care simulation research: extensions to the CONSORT and STROBE statements. Simul Healthc. (2016) 11:238–48. 10.1097/SIH.0000000000000150 27465839

[19] Agopian VG Markovic D Klintmalm GB Saracino G Chapman WC Vachharajani N. Multicenter validation of the liver graft assessment following transplantation (L-GrAFT) score for assessment of early allograft dysfunction. J Hepatol. (2021) 74:881–92. 10.1016/j.jhep.2020.09.015 32976864

[20] Robin X Turck N Hainard A Tiberti N Lisacek F Sanchez JC. pROC: an open-source package for R and S+ to analyze and compare ROC curves. BMC Bioinformatics. (2011) 12:77. 10.1186/1471-2105-12-77 21414208 PMC3068975

[21] Avolio AW Lai Q Cillo U Romagnoli R De Simone P. L-GrAFT and EASE scores in liver transplantation: need for reciprocal external validation and comparison with other scores. J Hepatol. (2021) 75:729–31. 10.1016/j.jhep.2020.12.009 33340580

[22] Tingle SJ Ibrahim I Thompson ER Bates L Sivaharan A Bury Y. Methaemoglobinaemia can complicate normothermic machine perfusion of human livers. Front Surg. (2021) 8:634777. 10.3389/fsurg.2021.634777 33598479 PMC7882904

[23] Pascher A Neuhaus P. Bile duct complications after liver transplantation. Transpl Int. (2005) 18:627–42. 10.1111/j.1432-2277.2005.00123.x 15910286

[24] Gowda S Desai PB Hull VV Math AA Vernekar SN Kulkarni SS. A review on laboratory liver function tests. Pan Afr Med J. (2009) 3:17. 21532726 PMC2984286

[25] Lee DD Croome KP Shalev JA Musto KR Sharma M Keaveny AP. Early allograft dysfunction after liver transplantation: an intermediate outcome measure for targeted improvements. Ann Hepatol. (2016) 15:53–60. 10.5604/16652681.1184212 26626641

[26] Golse N Guglielmo N El Metni A Frosio F Cosse C Naili S. Arterial lactate concentration at the end of liver transplantation is an early predictor of primary graft dysfunction. Ann Surg. (2019) 270:131–8. 10.1097/SLA.0000000000002726 29509585

[27] Abdul-Rahim AH MacIsaac RL Jhund PS Petrie MC Lees KR McMurray JJ.Efficacy and safety of digoxin in patients with heart failure and reduced ejection fraction according to diabetes status: an analysis of the Digitalis Investigation Group (DIG) trial. Int J Cardiol. (2016) 209:310–6. 10.1016/j.ijcard.2016.02.074 26913372

[28] Ali JM Davies SE Brais RJ Randle LV Klinck JR Allison ME. Analysis of ischemia/reperfusion injury in time-zero biopsies predicts liver allograft outcomes. Liver Transpl. (2015) 21:487–99. 10.1002/lt.24072 25545865

[29] Zhang QY Zhang QF Zhang DZ. The impact of steatosis on the outcome of liver transplantation: a meta-analysis. Biomed Res Int. (2019) 14:3962785. 10.1155/2019/3962785 31218224 PMC6536983

[30] Feng S Goodrich NP Bragg-Gresham JL Dykstra DM Punch JD DebRoy MA. Characteristics associated with liver graft failure: the concept of a donor risk index. Am J Transplant. (2006) 6:783–90. 10.1111/j.1600-6143.2006.01242.x 16539636

[31] Braat AE Blok JJ Putter H Adam R Burroughs AK Rahmel AO. The Eurotransplant donor risk index in liver transplantation: ET-DRI. Am J Transplant. (2012) 12:2789–96. 10.1111/j.1600-6143.2012.04195.x 22823098

