# Supplementary material for: Reassessment of Relevance and Predictive Value of Parameters Indicating Early Graft Dysfunction in Liver Transplantation: AST Is a Weak, but Bilirubin and INR Strong Predictors of Mortality
Source: Front Surg. 2021 Nov 16;8:693288. doi: 10.3389/fsurg.2021.693288 (PMC8634944; doi:10.3389/fsurg.2021.693288)
Supplement: Supplementary file 1 [file Data_Sheet_1.PDF]

Supplementary Material

Table S1: Control variables of cox proportional hazard model

Item	n	
Recipient Sex	616	
male		78% (478)
female		22% (138)
Recipient Age (Years/10) (median)	616	5.8 (IQR 1.3)
Body Mass Index (kg/m²) (median)	616	25.35 (IQR 5.75)
Donor Sex	616	
male		57% (352)
female		43% (264)
Donor Age (Years/10) (median)	616	5.2 (IQR 2.3)
Body Mass Index (kg/m²) (median)	616	25.70 (IQR 4.50)
MELD Score (mean)	616	21.41 (SD 9.18)
CHILD Score	616	
A		22% (137)
B		50% (308)
C		28% (171)
Acute Liver Failure	616	
no		96% (592)
yes		4% (24)
Surgical Risk Score	616	
none		62% (380)
low		22% (138)
middle		6% (34)
high		10% (64)
Re-Transplantation	616	
no		94% (577)
yes		6% (39)
Donor Risk Index (mean)	616	1.84 (SD 0.40)

MELD: Model of end stage liver disease

Table S2: Accuracies for patient and graft survival model

	EAD	AST	Bilirubin	INR	L-GrAFT	MEAF
**Patient**						
Wald test	2.84	0.31	12.64	6.82	-	4.32
Concordance	0.54 (SE=0.54)	0.51 (SE=0.51)	0.55 (SE=0.55)	0.51 (SE=0.51)	-	0.55 (SE=0.55)
C statistic (AUC)	0.527	0.501	0.537	0.511	-	0.528
Sensitivities	0.351	0.281	0.123	0.026	-	1.000
Specificities	0.703	0.721	0.952	0.996	-	0.002
**Graft**						
Wald test	7.00	3.19	13.17	14.97	54.55	11.39
Concordance	0.56 (SE=0.56)	0.54 (SE=0.54)	0.55 (SE=0.55)	0.52 (SE=0.52)	0.65 (SE=0.65)	0.57 (SE=0.57)
C statistic (AUC)	0.542	0.523	0.534	0.513	0.629	0.551
Sensitivities	0.371	0.314	0.114	0.029	0.993	1.000
Specificities	0.712	0.731	0.954	0.998	0.000	0.002

EAD: Early allograft dysfunction; AST: Aspartate aminotransferase; INR: International normalized ratio; MEAF: Model for Early Allograft Function; L-Graft: Liver Graft Assessment Following Transplantation; AUC: Area Under the Receiver Operating Characteristic

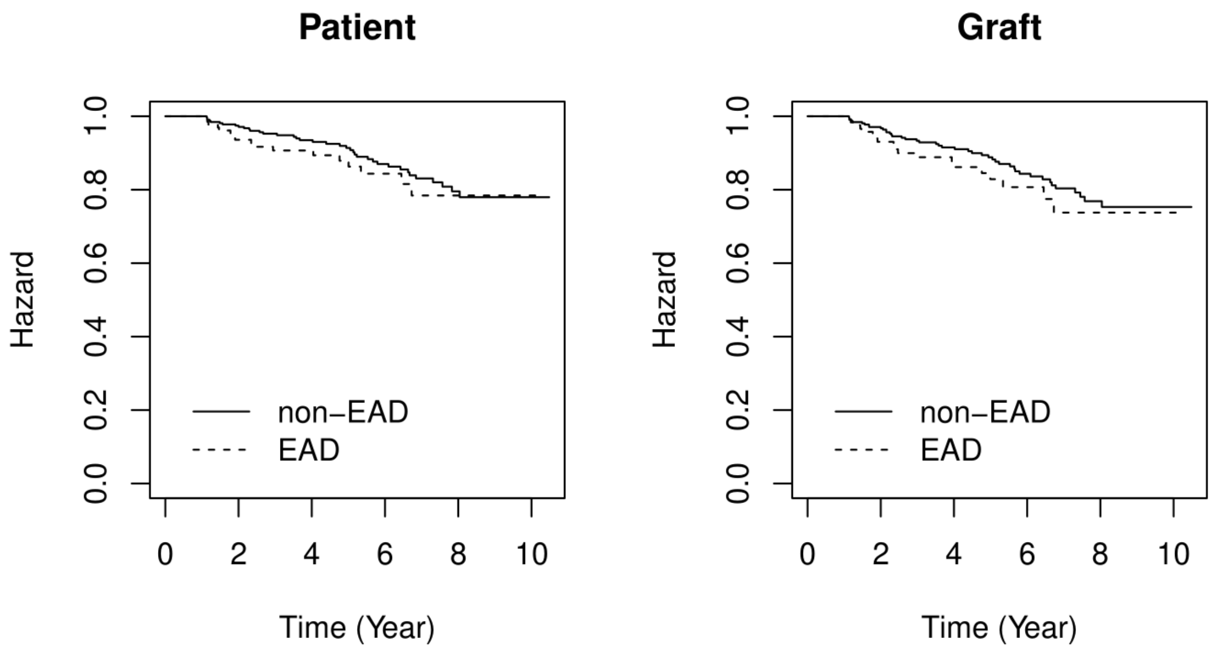

Figure S1: Patient and Graft survival by EAD status after 1 year

Difference in overall patient and graft survival between EAD and non-EAD groups after 1 year (*P*=.090; log rank-test=2.87; *P*=.008; log rank-test=7.13). Levels of significance: *P*-values < 0.05

EAD: Early allograft dysfunction

Patient Survival

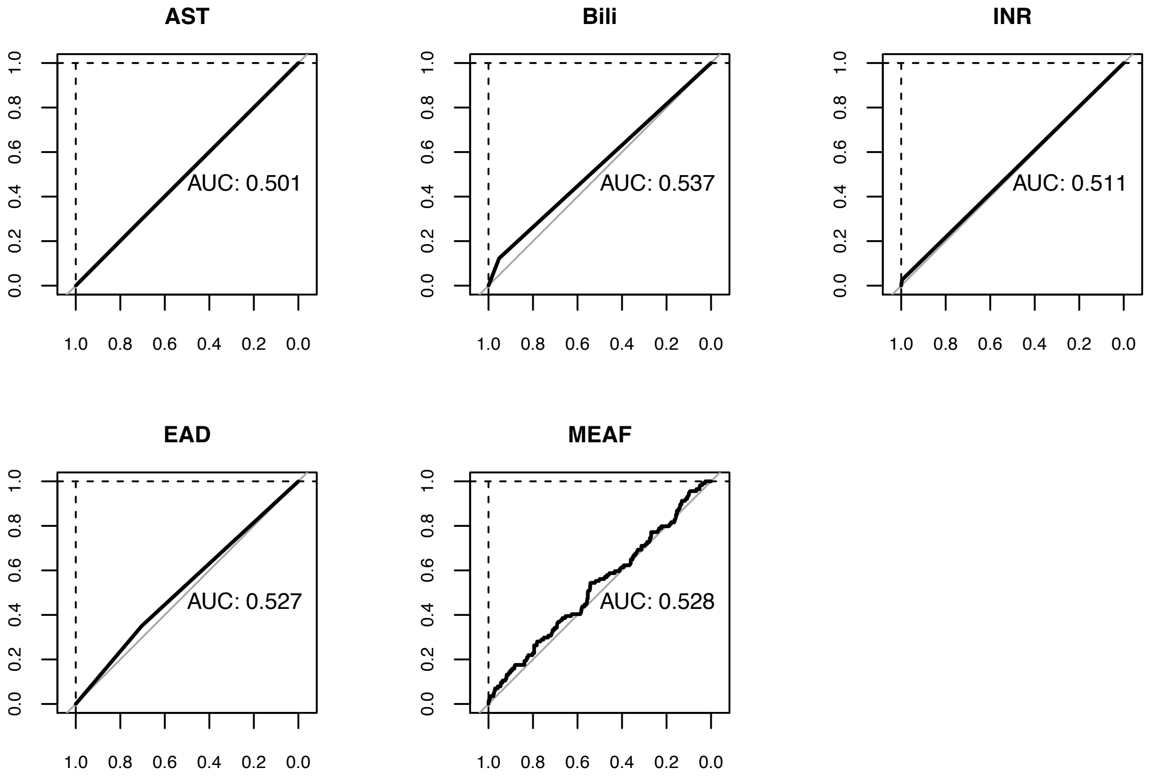

Graft Survival

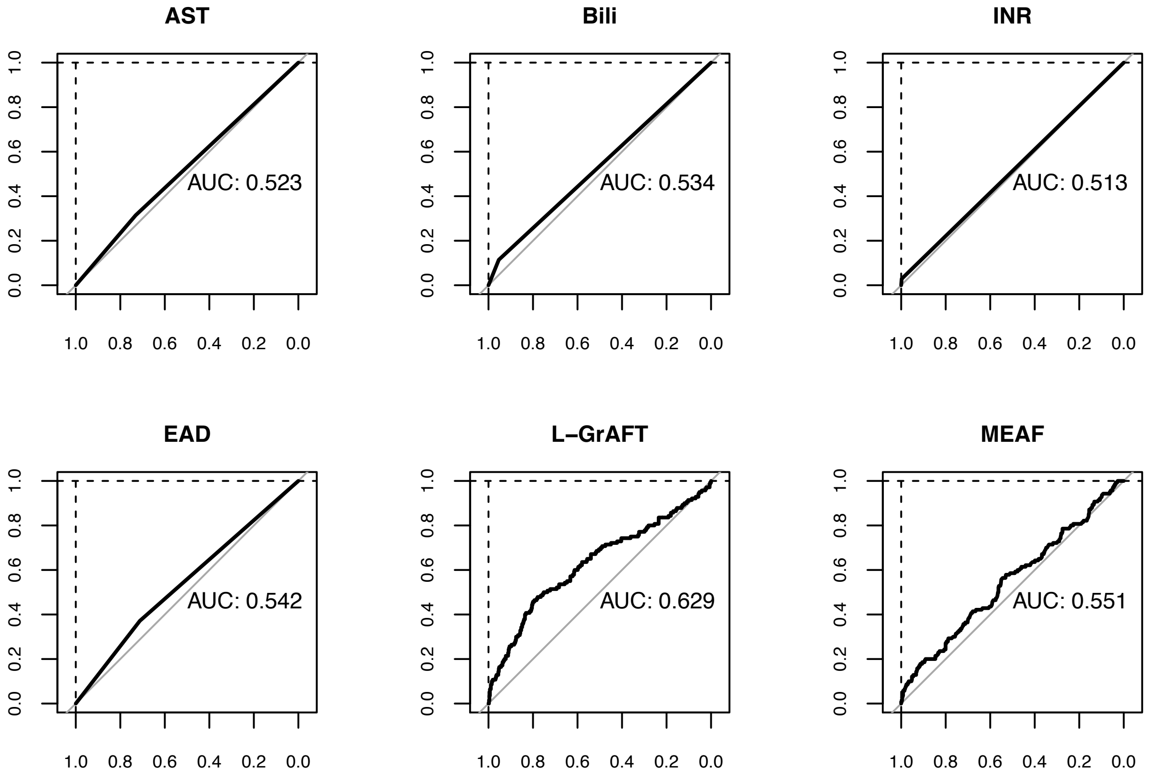

Figure S2: Comparison of Model Accuracies as Measured by the Area Under the Receiver Operating Characteristic (AUROC) Curve Among 3 Models of Early Allograft Dysfunction

AUROC of the Liver Graft Assessment Following Transplantation (L-GrAFT) score (C statistic .629), the Model for Early Allograft Function (MEAF) score (C statistic .528 and .551) and the early allograft dysfunction (EAD) score (C statistic .527 and .542)

EAD: Early allograft dysfunction; AST: Aspartate aminotransferase; INR: International normalized ratio; MEAF: Model for Early Allograft Function; L-Graft: Liver Graft Assessment Following Transplantation
